# Nano-Innovations in Cancer Therapy: The Unparalleled Potential of MXene Conjugates

**DOI:** 10.3390/ma17061423

**Published:** 2024-03-20

**Authors:** Sanjay Kulkarni, Soji Soman, Prerana D. Navti, Amrita Arup Roy, Ajinkya Nitin Nikam, P. Vineeth, Jahnavi Kulkarni, Krishnaraj Somayaji Shirur, Abhijeet Pandey, Sajan D. George, Srinivas Mutalik

**Affiliations:** 1 Department of Pharmaceutics, Manipal College of Pharmaceutical Sciences, Manipal Academy of Higher Education, Manipal 576104, Karnataka, India; sanjay987k@gmail.com (S.K.); sojineyyar@gmail.com (S.S.); prerananavti@gmail.com (P.D.N.); mamoamrita@gmail.com (A.A.R.); ajinkya.nikam7@gmail.com (A.N.N.); jahnaviljvg11@gmail.com (J.K.); abhijeet_pandey87@yahoo.com (A.P.); 2 Department of Atomic and Molecular Physics, Manipal Academy of Higher Education, Manipal 576104, Karnataka, India; vineethpnair96@gmail.com (P.V.); sajan.george@manipal.edu (S.D.G.); 3 Department of Conservative Dentistry and Endodontics, Manipal College of Dental Sciences Manipal, Manipal Academy of Higher Education, Manipal 576104, Karnataka, India; sk.somayaji@manipal.edu

**Keywords:** MXene, 2D materials, conjugates, cancer, nanotheranostics

## Abstract

MXenes are two-dimensional transition metal carbides, nitrides, and carbonitrides that have become important materials in nanotechnology because of their remarkable mechanical, electrical, and thermal characteristics. This review emphasizes how crucial MXene conjugates are for several biomedical applications, especially in the field of cancer. These two-dimensional (2D) nanoconjugates with photothermal, chemotherapeutic, and photodynamic activities have demonstrated promise for highly effective and noninvasive anticancer therapy. MXene conjugates, with their distinctive optical capabilities, have been employed for bioimaging and biosensing, and their excellent light-to-heat conversion efficiency makes them perfect biocompatible and notably proficient nanoscale agents for photothermal applications. The synthesis and characterization of MXenes provide a framework for an in-depth understanding of various fabrication techniques and their importance in the customized formation of MXene conjugates. The following sections explore MXene-based conjugates for nanotheranostics and demonstrate their enormous potential for biomedical applications. Nanoconjugates, such as polymers, metals, graphene, hydrogels, biomimetics, quantum dots, and radio conjugates, exhibit unique properties that can be used for various therapeutic and diagnostic applications in the field of cancer nanotheranostics. An additional layer of understanding into the safety concerns of MXene nanoconjugates is provided by detailing their toxicity viewpoints. Furthermore, the review concludes by addressing the opportunities and challenges in the clinical translation of MXene-based nanoconjugates, emphasizing their potential in real-world medical practices.

## 1. Introduction

**Figure 1:**
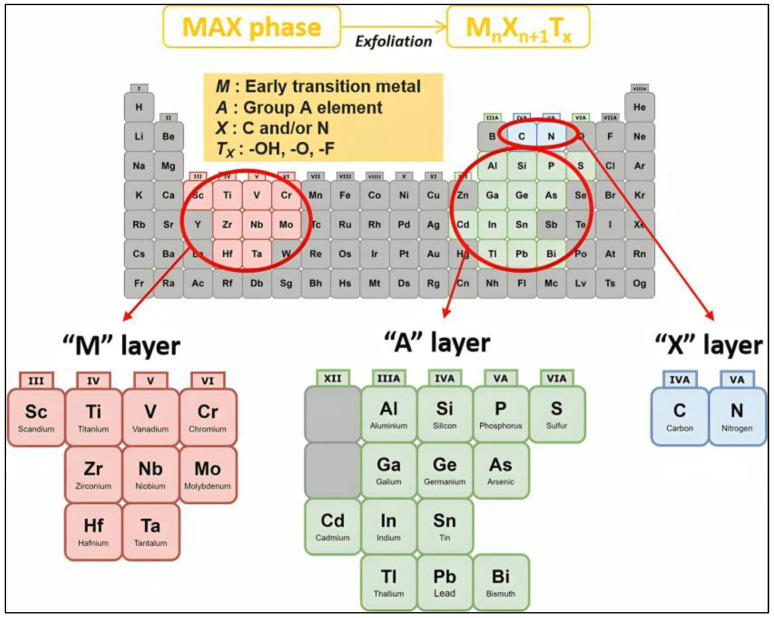
General element composition of MAX phase and MXene: M: early transition metal; A: Group A element; X: C and/or N; Tx: surface functional group. Reproduced with permission from [10]. Copyright © 2021, Springer Nature.

MXenes are a family of 2D materials that have attracted significant interest in the field of materials science since their discovery [1]. They are derived from a class of layered ternary compounds called MAX phases, which consist of transition metals such as carbides, nitrides, or carbonitrides. The MAX phases follow the standard formula for M_n+1_AX_n_. Here, “M” denotes early d-block transition metals, “A” represents the main-group sp elements (mostly IIIA or IVA), and “X” signifies carbon and/or nitrogen atoms (Figure 1) [2,3]. Their unique properties, which result from a rare blend of ceramic and metallic behaviors, pique great attention. MAX phases exhibit low density, high hardness, and great corrosion resistance in ceramics, while high thermal and electrical conductivities and outstanding machinability are comparable to those of metallic materials. Because of these qualities, MAX phases are attractive materials for high-temperature structural applications. MXenes are very attractive, being at the same level as transition metal dichalcogenides (TMDs) or metal oxides, or even better in some aspects such as having strong bonds that inhibit cleavage through shearing or other mechanical techniques [4]. Furthermore, compared with metal oxides and TMDs, MXenes are more tunable and potentially more useful for energy storage applications [4]. More than 150 MAX phases have been reported to date [5]. Over the years, the process of synthesizing MXenes has undergone several substantial advancements, leading to the creation of multiple techniques and etching conditions that produce precise MXene compositions, surface chemical information, and flake sizes [6]. Researchers have broadened the synthesis of MXenes, progressing from traditional wet-chemical etching to more advanced methods such as molten salt and electrochemical etching [7]. MXenes are extremely versatile materials with a wide range of applications, including mechanical energy conversion, energy storage, drug delivery, water purification, tissue engineering, regenerative medicine, and conductive coating [8]. MXenes have been researched as nanozymes, and they have several benefits, including affordability, stability, and strong catalytic activity [9]. Ongoing research has revealed the immense potential of MXenes in various fields due to their exceptional properties.

Owing to their adjustable morphology, biocompatibility, and ease of functionalization [11], MXenes possess good potential for fabricating MXene-based composites, where they are incorporated with other materials to benefit from their combined properties. This amalgamates the distinctive properties of MXenes and those of partner materials, offering better functionality and performance [12,13,14,15]. The exploration of MXenes continues to expand in medical science, with ongoing research focused on synthesis methods, fundamental properties, and novel biomedical applications. Cancer is a broad and complicated category of disease characterized by the uncontrolled growth and division of aberrant cells that can invade and spread throughout the body [16,17]. This disease appears in a variety of ways, with genetic abnormalities, environmental exposures, and lifestyle decisions all contributing to its beginning. Our understanding of cancer has evolved over time, from ancient ideas linking it to bodily imbalances to today’s recognition of its multiple characteristics, which includes research into genetic complexities and the function of the immune system. Despite breakthroughs in treatment methods, identifying targeted and effective medicines remains an important task, necessitating novel approaches in the field of cancer [18,19]. In recent years, the advent of two-dimensional (2D) materials has inspired significant interest in cancer research. The ultrathin architectures, unique physicochemical features, and large surface areas of these materials have shown significant potential for cancer diagnosis and therapy. Graphene and black phosphorus are well-known 2D materials with high biocompatibility and conductivity, making them promising candidates for targeted drug delivery and imaging [20,21,22]. The use of 2D materials for cancer research is a new frontier in which nanotechnology can potentially transform the approach to cancer detection, therapy, and monitoring. On the basis of our previous publication on MXenes [23], we discuss the various biomedical applications of MXenes in detail; however, the current article comprehensively explores the properties of MXenes and their use in fabricating MXene conjugates for cancer nanotheranostics. In this review article, we shed light on the potential of different MXene conjugates, including polymers, metals, graphene, and other conjugates, as promising candidates for advancing cancer diagnosis and therapy. This article also highlights areas requiring further research and development, providing an overview of the research gaps in exploring the potential of MXene conjugates for cancer theranostics. Additionally, this review delves into relatively unexplored areas of MXene conjugate synthesis for biomedical applications, particularly in cancer management. Furthermore, this study examined the characteristics and functionalization of MXene conjugates while highlighting biosafety and stability concerns. This article also highlights areas requiring further research and development, providing an overview of the research gaps in exploring the potential of MXene conjugates for cancer theranostics.

## 2. Synthesis of MXenes

The MXene synthesis technique impacts the physicochemical features, electrical features, and number of applications. The approaches utilized for MXene synthesis include etching, top-down methods, sublimation methods, hydrothermal methods, and bottom-up methods. These are discussed below in detail.

### 2.1. Top-Down Approach

**Figure 2:**
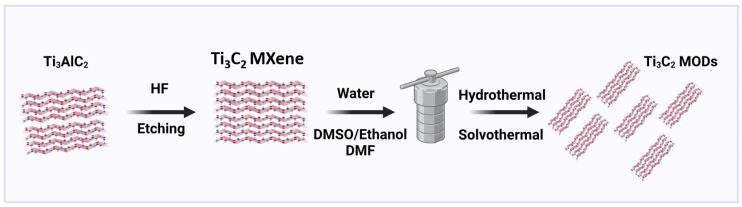
Schematic illustration showing the etching and solvothermal synthesis of MXenes.

Graphene, WS_2_ powder, carbon nanotubes, MoS_2_ crystals, black phosphorus, and other 2D and 3D precursors have been converted to nanosheets and quantum dots using a top-down approach [24]. This process involves exfoliation and delamination of the bulk MAX phases to few-layered MXene sheets. This method is important because it can also be performed at lower temperatures. Large-scale production can be achieved by utilizing many raw materials via this technique. Nevertheless, this method has several drawbacks, including its limited yield and requirement for particular treatments. The three most popular top-down methods are acid refluxing, hydrothermal treatment, and ball milling. The solvothermal method involves the reaction of raw materials dissolved in a solvent and heating in an autoclave above the boiling point of water. The combined effects of pressure, solution pH and high temperature could be utilized to alter the shape, size, and morphology of MXenes [25]. The solvothermal process is a popular choice for preparing MXenes since it is more effective than the hydrothermal method. Figure 2 describes MXene synthesis by the HF etching method followed by its conversion to quantum dots (QDs) using the solvothermal method. In this method, temperature and the choice of solvent play crucial roles. For instance, Peng et al. synthesized Ti_3_C_2_ MXene using a hydrothermal method with the lower toxicity etching agents NaBF_4_ and HCl. Compared to the Ti_3_C_2_ produced through HF etching, the Ti_3_C_2_ MXene synthesized using the hydrothermal technique exhibited a greater c-lattice parameter, wider interlayer spacing, and larger BET specific surface area. This is because the hydrothermal process involves a gradual release mechanism. This approach not only avoided the use of higher HF concentrations but also efficiently prepared Ti_3_C_2_ flakes [26]. Barkha Singh and coworkers reported a Ti_3_C_2_ MXene nanobipyramid (Ti_3_C_2_NB) obtained by a three-step procedure that included exfoliation, delamination, and hydrothermal treatment. The Ti_3_C_2_NB exhibited fluorescence along with excitation-dependent emission. The MXene nanoprobyramids showed a 43% increase in photoluminescence intensity with increasing pH from 3 to 7 and a 38% increase with increasing temperature from 20 to 80 °C. These Ti_3_C_2_NB materials showed better biocompatibility. These materials can be used as sensors for temperature and pH and in bioimaging [27].

Another approach is the molten salt etching process (MSE). The MSE approach possesses a faster processing time (30 min). Here, MXenes are prepared by heating MAX phases such as Ti_4_AlN_3_ in a molten fluoride salt mixture (i.e., NaF, LiF, and KF (12:29:59 weight ratio)) at 550 °C under Ar shielding to obtain Ti_4_N_3_. Extra cleaning by washing with deionized water and H_2_SO_4_ and delamination in TBAOH is mandatory. The crystallinity of the delaminated Ti_4_N_3_ MXene is less than that of the MXene obtained by HF etching, which was confirmed by the XRD patterns of Ti_4_N_3_. The MXenes obtained by this method possess the disadvantages of low crystallinity, low purity, surface defects, and high energy and heat consumption [24]. Urbankowski and coworkers used a mixture of molten NaF, LiF, and KF salts for 30 min at 550 °C for etching Ti_4_AlN_3_ to obtain nitride MXenes [28]. It is very difficult to develop nitride MXenes by methods other than molten salt etching. HF-etched Mo_2_C can be transformed to Mo_2_N MXene via heat treatment at 600 °C in an ammonia atmosphere [24]. Liu and coworkers recently reported MS-Ti_3_C_2_T_x_ synthesis by the intercalation of TBAOH and subsequent delamination by sonication. The obtained MS-Ti_3_C_2_T_x_ with a -Cl termination was used as an anode in a Li-ion battery, achieving a high specific capacity and good rate capability [29]. A nitride-based MXene was prepared using the MSE approach. In this method, a molten fluoride salt mixture was mixed with inert Ar gas at 550 °C to etch the Al layer from Ti_4_AlN_3_. Delamination was performed by TBAOH to produce Ti_4_N_3_T_x_ MXene monolayers [28]. Alternatively, larger single flakes of MXene can be isolated using the minimally intensive layer delamination (MILD) method without sonication, resulting in less defective flakes [30]. The MILD approach has improved research prospects for electrical, optical, and size-dependent material properties while also improving scalability. The MILD process requires centrifugation to remove large agglomerates and unexfoliated MXene particles. During centrifugation, the dispersed MXene solution is spun at high speeds, causing the heavier particles to settle to the bottom of the centrifuge tube. The authors recommended using the MILD approach in applications requiring larger flake sizes, high electrical conductivity, and environmental stability.

Most of the synthesis methods for MXenes require fluoride-based chemicals or HF, which can form -F and -O terminations at the MXene interface. The -F terminations reduce the electrochemical performance of MXene-based supercapacitors. Hence, fabrication methods free from fluoride are required to provide favorable electrochemical properties [24]. Chen et al. utilized a simple and time-saving electrochemical etching approach to prepare -Cl-containing and -F-free Ti_3_C_2_T_x_ in mixed LiCl and LiOH solutions. The method showed an etching efficiency of 92.2%. For delamination, sonification alone was used without the use of any organic intercalant [31]. Yang et al. adopted a redox coupling method involving a Lewis acid molten salt cation and the A element for etching the MAX phases. Fluorine-free Ti_3_C_2_ MXenes with -I, -Br, and -Cl were developed. The developed Ti_3_C_2_I_2_, Ti_3_C_2_Br_2_, and Ti_3_C_2_Cl_2_ showed pseudocapacitive-like behavior in H_2_SO_4_ (3 M) and distinguishable charge–discharge rates. These excellent super capacitive features of Ti_3_C_2_-MXenes as electrodes with aqueous electrolytes are due to the higher electrochemical activity of Br, I, or Cl [32].

The ball milling approach has been utilized for fabricating various QDs as a top-down method to reduce nanoparticle (NP) size. The morphological and physical attributes of the formed products are affected by many factors, including the milling speed, milling type and duration, and ball-to-powder weight ratio [33]. Zhang and coworkers utilized this method for fabricating Ti_3_C_2_T_x_ nanodots by mixing Ti_3_C_2_T_x_ and several solid-state precursors, such as C, S, P, and Si. The powder-to-ball ratio was approximately 1:20, and the BM was incubated at 550 rpm for 2 days in an Ar atmosphere. Red-P-aided ball milling leads to the formation of QDs with a diameter in the 6–25 nm range [33]. An ultrasonic approach is another environmentally friendly method used for the preparation of MXenes. The ultrasonication technique converts both nonlayered and layered materials into QDs owing to the reverberation and acoustic cavitation of the solvents. The delamination, separation, and exfoliation of MXene sheets are important steps for producing high-quality 2D sheets. Ultrasonication greatly affects the structure and dimensions of MXene materials. This process can generate MXenes with a diverse range of particle sizes and morphologies [34]. Ultrasonic irradiation efficiently separates the MXene flakes, producing MXenes with lateral dimensions ranging from 0.2 to 1 µm and thicknesses less than 10 nm. Under different ultrasonication conditions, MXene particles can develop various shapes, including plate, thin sheet, crumpled, spherical, and scroll morphologies. Improper sonication can lead to impurities and flaws. The choice of solvent for ultrasonication can affect the delamination performance, particle size, morphology, and concentration of MXene materials. Solvents with elevated surface energy boiling points, such as DMF, NMP, DMSO, and TBAOH, can effectively counteract the binding forces of the bulk and convert them into small QDs [35].

### 2.2. Bottom-Up Approaches

Unlike the top-down approach involving bulk material as the starting material, the bottom-up approach utilizes molecular materials as the starting material. Employing this method, MXene quantum dots can also be developed from small precursors of inorganic and organic molecules. This technique has many advantages, such as fast functionalization, increased atomic usage, and morphological and structural control, giving MXenes excellent properties and structures [24]. Owing to the simple operating conditions in comparison to those of top-down methods, a bottom-up approach could be utilized in the future for preparing MXenes since there is limited research available on this approach. The most widely used bottom-up approach is pyrolysis [24]. The process involves heating the raw materials at higher temperatures in inert atmospheres to decompose the A layer and form MXenes. However, the limited yield and the requirement for specific treatments are the disadvantages of this technology. A group of researchers have prepared a quasi-MXene structure C/Fe_3_C/Fe composite by employing a simple pyrolysis procedure. The importance of ionic liquids in regulating product morphology was explored, and a probable mechanism was proposed. The quasi-MXene hybrid was found to possess excellent electrochemical performance and a longer lifespan than other hybrid materials [36]. Wang and coworkers developed Mo_2_C QD–carbon polyhedron hybrids by employing a pyrolysis approach using zinc acetate, 2-methylimidazole and molybdic acid as starting materials [37]. A higher pyrolysis temperature of the MXene/graphene polymer hybrid resulted in destruction of the electromagnetic interference (EMI) shielding process [38]. Therefore, in bottom-up methodologies, alterations in reaction parameters, such as reaction temperature and duration, starting materials (precursors), and concentration, play crucial roles in the development of MXenes. This method is straightforward and efficient and exhibits monodispersity in contrast to the top-down approach. Nevertheless, it is imperative that these techniques be further considered to effectively satisfy unfulfilled requirements.

Another method used in the bottom-up approach is chemical vapor deposition (CVD), which involves the introduction of a gaseous precursor or a combination of precursors into a chamber. These substances then undergo a reaction under carefully regulated circumstances, resulting in the deposition of a solid material onto a surface. The reaction often entails the breakdown of gaseous precursors, resulting in the creation of a thin layer on the substrate. Wang et al. [39] reported direct synthesis for scalable and economic preparation of MXenes by reacting metal halides or metals with methane, nitrogen, or graphite. This type of direct synthesis procedure allows CVD growth of MXenes and spherulite morphologies. Chuan Xu et al. developed defect-free and high-quality ultrathin crystals of molybdenum carbide employing a CVD procedure. These crystals exhibited 2D superconductivity at low temperatures with sizes up to or larger than 100 μ [40]. However, its limitations include the production of hazardous byproducts, the need for specialized equipment, and issues relating to material quality and scalability based on the choice of metal catalysts.

## 3. Properties of MXenes

The properties of MXenes include improved thermal and electrical properties, an adjustable electronic band gap, and a higher Young’s modulus. MXenes also possess magnetic, electronic, mechanical, vibrational, and electrochemical properties. MXenes possess high metallic conductivities and hydrophilic surfaces, which differentiates them from graphene and other 2D materials. The performance and properties of MXenes can be modified via (i) surface functionalization (by thermal and chemical treatments) and (ii) composition (different transition metals, “M” and “X”, solid solution formation) [41,42]. It is crucial to understand and identify the properties of MXenes, which in turn lays the foundation for fabricating MXene conjugates in which MXenes with different properties can be amalgamated. In this section, a detailed overview of the different properties of MXenes and their possible applications in the biomedical field are given.

### 3.1. Electronic and Transport Properties

Compared with those of MAX phases, the electronic and transport properties of MXenes are altered by alterations in solid solution formation, stoichiometry, and functional groups [43]. According to the experimental results, the electrical conductivities of the MXene pressed discs were greater than those of reduced graphene oxide materials, and the electrical conductivities were analogous to those of multilayered graphene [44]. The resistance of MXenes relies upon the layers and the types of functional groups. It increases with increasing layer length [41]. Ti_3_C_2_T_x_ showed electrical conductivities ranging from 850–980 S m^−1^ because of the differences in (i) surface functional groups, (ii) defect concentrations, (iii) d-spacings among MXene flakes, (iv) delamination yields, and (iv) lateral sizes induced by each etching process [44]. A lower concentration of HF and shorter etching time led to the formation of MXenes with fewer defects and greater size, resulting in greater electrical conductivity. Larger Mxenes have shown 5-fold greater conductivity than small Mxenes. The conductivities of Mxenes could also be affected by the background humidity, resulting in their application in humidity sensing.

Mxenes display various electronic features, including semiconducting, semi metallic, and metallic phases. Notably, Ti_3_C_2_T_x_ Mxene exhibits outstanding metallic behavior, demonstrating its effectiveness in detecting ultralow gas concentrations (e.g., acetone: 50–1000 ppb; ammonia: 100–1000 ppb) with a signal-to-noise ratio two orders of magnitude greater than that of other 2D materials [45]. Mxene-based RRAM devices have exceptional bipolar resistive switching properties, such as a high ON/OFF ratio of 10,000 and a long retention period (5 × 10^4^ s), due to the thin atomic layers of Mxenes [46]. Moreover, Mo_2_CT_x_ Mxene thin-film photodetectors demonstrate a significant photocurrent in the 400–650 nm wavelength range, attaining a maximum on/off ratio of approximately 200 under 660 nm light. These results underscore their remarkable photodetection capabilities, stability under continuous illumination, and durability against repeated mechanical stress [47].

Surface modification by alkaline processes is vital for enhancing the thermal and electronic properties of Mxenes [44]. These surface-modified composites exhibit an increase in conductivity of up to 2-fold. This increase in conductivity is due to the removal/alteration of functional groups (especially -F) and intercalated molecules [41]. Many studies on the electrical properties of Ti_3_C_2_ have focused on the modification of surface terminations by thermal treatment. However, a further increase in the calcination temperature above 800 °C led to the collapse of the Ti_3_C_2_ nanosheets and destruction of the 2D nanostructure [48]. Another method for altering the electronic conductivity of Mxenes involves the use of reinforcing materials. An example of such a material is chitosan. Herein, with increasing chitosan concentration, the electrical resistance of the respective Mxene also increased [49]. To understand the mechanism by which some Mxenes become semiconductors after functionalization, researchers have explored both pristine and functionalized Mxene structures. The results showed that the Mxenes are analogous to bare MAX phases and are metallic owing to the Fermi energy generated by the M d orbital. A small band gap between the *p* and *d* bands of the X-ray *p* band is present somewhat beneath the *d* band. The functionalization with F led to a downwards shift in the Fermi energy owing to the gain of one electron from the system by each -F, whereas O functionalization led to additional downwards shifting, allowing Ti_2_O_2_ to act as a semiconductor [50,51]. This property of Mxenes is widely utilized in energy storage applications; nevertheless, Mxenes can also be employed in biological research due to their exceptional electrical properties. For instance, the electrical conductivity of Mxenes changes when biomolecules are bound to their surface, enabling the identification of biomolecules. Additionally, Mxenes can be utilized in electronics for monitoring physiological signals from the body [52].

### 3.2. Optical Properties

In recent years, numerous optical properties, such as plasmonic behavior, scattering, emission, photoluminescence, nonlinear refractive indices, saturation absorption, optical transparency, and effective photothermal conversion, have been demonstrated for MXenes. The interaction of MXenes with light in different ways has an important influence on MXene research [53]. Surface terminations play a vital role in regulating the optical properties of MXenes. Muhammed and coworkers determined the effect of M (V or Ti) and surface terminations (such as F and O) on the plasmonic and optical features of M_2_CT_2_ MXene nanoflakes. These authors revealed the development of localized surface plasmon resonance (LSPR) at a very low energy level in the infrared (IR) region, an important region specific for chemical sensing, biological imaging, optical communication, heat regulation, and chemical sensing. The Janus MXene nanoflake Ti_2_CFO (with a -F top surface termination and a -O bottom surface termination) shows the narrowest and strongest IR LSPR owing to its large time-dependent out-of-plane dipole moment. These M_2_CT_2_ MXene nanoflakes indicate the potential for plasmonic behavior. These study results could be helpful for exploring MXene plasmonics [54].

Usually, MXenes exhibit very low photoluminescence (PL) in aqueous solution. To widen the applicability of MXene-QDs (MQDs), especially in the optical and biological arena, the formation of QDs is a suitable technique because edge effects and quantum confinement can occur when QDs are thin. Unlike nanosheets, QDs are very small and possess luminescence [55,56]. The PL of 2D nanosheets of MXenes is less than that of QDs [57]. In addition to the QDs obtained from traditional materials, a few MXene-based QDs with PL characteristics, such as V_2_C MQDs and Ti_3_C_2_, have also been reported, among which the latter is the most researched [55,56]. Reports have suggested that MQDs can be prepared by cutting bulky Ti_3_C_2_ MXene layers via a hydrothermal approach with a quantum yield of up to 10%. Colloidal MQDs with various morphologies, such as MQD-100, MQD-120, and MQD-150, can be obtained. All the MQDs obtained were found to possess luminescence, but MQD-150 was found to have greater cytotoxicity than the other two products; hence, it was unsuitable for biomedical applications [58]. Some properties of MXenes that are useful for industrial use include saturable MXene absorption, which is appropriate for ultrafast laser applications. The MXene Ti_3_C_2_T_x_ exhibited a wide range of wavelengths, specifically spanning from 1550 to 1620 nm. This range encompasses the C-band, which is a significant telecommunication band utilized for communication and signaling purposes [59]. The efficient nonlinear absorption coefficient was −10^−21^ m^2^/V^2^, indicating its usefulness in optical switching areas [60,61]. Ti_3_C_2_ MXenes have shown efficacy in imaging, diagnosis, and therapeutic monitoring. Hence, MXenes could be employed for synergistic therapy, facilitating lesion monitoring. Mxenes possess various applications in sensing, photocatalysis, photothermal treatment, photoacoustic imaging, photodynamic therapy, biosensing, and controlled drug release. Hence, MXenes have been studied for use in tumor ablation and cancer therapy. Owing to their surface plasmon resonances, they display efficacious near-infrared light absorption and intense photothermal conversion [62].

### 3.3. Magnetic Properties

Materials with controllable and strong magnetic moments are essential for application in spintronics. For most MXenes (bare or otherwise), the ground states are nonmagnetic. This is due to the very strong bonding between the X element and the transition metal. However, several MXenes, including Ti_2_N^52^ and Cr_2_C^51^ (ferromagnetic) and Mn_2_C^54^ and Cr_2_N^53^ (anti-ferromagnetic), are predicted to possess intrinsic magnetism. The reason for this magnetism could be (i) defects in the monolayers, (ii) the innate characteristics of the transition metal, and (iii) surface terminations [53]. However, the magnetic properties of MXenes have been less explored. The theoretical predictions differ from the actual practical experimental results. The predicted magnetic MXenes include magnetic transition metals (Mn, V, Cr, Mo, Fe, Ni, and Co) or their doped configurations [63]. Recently, magnetism was revealed in reduced Ti_3_C_2_. This is the first report on magnetic MXenes. The magnetic sensitivities are temperature-dependent beyond a temperature of ~10 K, suggesting that the reduced Ti_3_C_2_ is Pauli paramagnetic, whereas a Curie-like enhancement at temperatures <10 K indicates that magnetism emerges from 2D layers rather than from the reduced TiO_2_ NPs. In conclusion, the reduction of Ti_3_C_2_T*_x_* enhanced its paramagnetic properties. These reduced MXenes could have various applications in magnetic devices [64].

The magnetic properties of MXenes are highly susceptible to the surrounding environment. Ly and his colleagues have shown that a monolayer of Ti_2_C changes from a ferromagnetic, half-metal, magnetic metal, or nonmagnetic semiconductor to an AFM semiconductor when an electric field is applied. Additionally, an electric field strength above a specified threshold leads to a significant decrease in the magnetic moments of titanium atoms, which results in a reduction in the effective mass of the atoms. Thus, modifying the external electric field can alter the magnetic characteristics of MXenes [65]. MXenes possess electromagnetic interference shielding (EMI-S) related to the X-band that exceeds 70 dB at a thickness of 0.8 mm, making them highly effective EMI-S materials. Owing to the multilayered architecture of MXenes, the absorption efficiency is increased, and the internal electromagnetic attenuation is strengthened. Due to their higher electrical conductivity, a high amount of electromagnetic reflection could also occur on the surface. Due to these magnetic properties, MXenes could be useful as contrast agents in bioimaging. However, further improvements in the shielding abilities of electromagnetic interference are required to determine the usefulness of these materials in biomedicine [57].

### 3.4. Mechanical Properties

Although MXenes possess flexible characteristics 2–3 times less than those of graphene, they possess a bending stiffness ~3.5 times greater than that of graphene (1.2 eV for graphene vs. 4.47 eV for Ti_2_C), demonstrating that these materials could be utilized as composite strengthening materials [66]. These materials exhibit superior contact potential compared to graphene in hybrid applications due to their inherent inclusiveness. The atomic layer thickness is responsible for the mechanical flexibility of MXenes. The physical characteristics of MXenes might differ depending on the surface termination. Bai et al. demonstrated a significant connection between Ti atoms and O terminations in Ti_3_C_2_ and Ti_2_C, in contrast to the interaction observed with OH- and F-terminated MXenes. In addition, MXenes with O terminations have exceptional rigidity because of the strong bonding between Ti and O (607 GPa), surpassing the binding strength of titanium–fluorine (Ti-F; 391 GPa) and titanium–hydroxyl (Ti-OH; 285 GPa) bonds [67]. Yorulmaz and coworkers calculated the Young’s modulus and demonstrated that the MXene becomes stiffer with increasing transition metal mass. This finding was not the same for nitrides [68]. Several MXenes based on carbides exhibit mechanical stability and have greater elastic moduli than MXenes made of nitrides. Moreover, individual MXene flakes exhibit instability in settings containing oxygen and moisture, yet they remain stable in water when oxygen is eliminated or in dry air. Numerous investigations [69,70,71] have shown that when exposed to air and water, Ti_3_C_2_T_x_ MXene progressively oxidizes into titanium dioxide and carbon, resulting in a decrease in its stability and performance. It has been discovered that dissolved oxygen plays an important role in the breakdown of MXene nanosheets in water [71]. The main oxidation mechanism is driven through hydrolysis [72,73]; thus, it is evident that isolating air and water may impede the oxidation and hydrolysis of MXenes. The few available approaches for improving the stability of MXenes include defect passivation, the use of organic dispersions, and the use of polymer composites [74]. Polymers such as PVA and PVP have been shown to improve the stability of MXenes by decreasing the number of reaction sites. However, these polymer composites do not completely prevent oxidation. For instance, the conductivity of a sample with 50 wt% filler (PVA) decreased to 20% of the initial value after 50 days [75]. This requires a greater filler content to attain the necessary performance, which increases the cost and degrades the material’s mechanical qualities. Polystyrene (PS)/MXene composites have also been reported [76] to improve MXene stability. PS/MXene composite particles form a 3D conductive network structure that resists oxidation and enhances electrical conductivity. The results showed that the compact and ordered 3D conductive network structure has greater performance stability than the usual random structure. The conductivity of the composite with a filler (PS) content of 1.81 vol% maintained 53.4% of the initial value after 180 days. Surface modification of MXenes with soybean phospholipids [77], polyethylene glycol (PEG) [78], or cellulose hydrogels [79] has been shown to increase their stability in anticancer applications. However, detailed investigations addressing the stability of these materials are lacking. To address this issue, a study [80] was conducted on the surface modification of MXenes utilizing dopamine (DA) and sodium ascorbate (SA) to improve their stability and photothermal therapy (PTT) against cancer. DA was chosen for its antioxidative characteristics due to the presence of catechol moieties, while SA was chosen to decrease surface oxidation and promote colloidal stability. The storage stability of surface-modified MXenes was explored by comparing their UV–Vis spectra, and it was discovered that the absorbance spectra of surface-modified MXenes after 40 days of storage were identical to those obtained after 0 days of storage. However, due to their instability, the unmodified MXenes showed a decrease in NIR absorbance after 40 days of storage compared to 0 days.

## 4. Fabrication of MXene Conjugates

MXene conjugates are functionalized materials made of MXene that have been combined or chemically altered for a variety of applications. These conjugates can be engineered to display particular characteristics or abilities according to their intended use. Surface nanopore fabrication techniques have the potential to enhance factors such as drug delivery capabilities, hydrophilicity, stability/dispersion, biocompatibility, and mesoporous structural characteristics of MXenes. However, to address concerns related to toxicity and structural defects, additional research is needed to optimize surface modifications as well as multifunctionalization strategies. Furthermore, comprehensive studies are required to investigate factors such as biocompatibility, biodegradability, biodistribution, immune system interactions, controlled delivery, and sensitivity to neurotransmitters for potential clinical and biomedical applications [81]. MXene conjugates can be fabricated through a range of methods, including hydrothermal synthesis, deposition techniques, hot pressing, drop casting, solution processing, and in situ polymerization. These methods allow for the creation of customized nanocomposites that possess improved properties for use in energy storage, catalysis, sensing, and biomedical engineering. Each method of MXene fabrication has its own advantages and disadvantages. It is important to prioritize investigating one-step greener synthesis techniques that are cost-effective, safe, and efficient. The focus should be on optimizing conditions to fabricate multifunctional nanostructures that produce in high yields and exhibit good biocompatibility [82]. Table 1 details the different fabrication techniques used for the preparation of MXene conjugates. This table shows the numerous methods for producing MXene conjugates and their applications. The adaptability of MXene conjugates, as illustrated by the numerous manufacturing processes and uses outlined in the table, emphasizes their potential to address a wide range of disciplines.

### 4.1. Hydrothermal/Solvothermal Synthesis

The synthesis of MXene nanocomposites with enhanced physiochemical and thermomechanical properties using the hydrothermal/solvothermal method, a chemical strategy, is a cost-effective and straightforward process [83]. The reaction involved mixing the mineralizer, liquid solvent, and precursor substance at high pressure and temperature for a specific time and then washing. Nanocrystals produced through hydrothermal/solvothermal processes exhibit excellent properties, such as strong crystallinity, good morphology, precise NP size control, and outstanding dispersibility [15]. Therefore, this method is widely used to produce controlled MXene-based nanocomposites. The hydrothermal method uses water as the solvent, while organic solvents are employed in the solvothermal method [84]. By subjecting the reaction mixture to high temperature and pressure using an autoclave, a supercritical fluid is generated with increasing temperature and pressure above the critical point of the solvent, allowing for efficient dissolution of chemical compounds that are insoluble under normal conditions [85,86]. However, a significant drawback of this method involves the formation of corrosion by hydroxyl units in the presence of high temperature and pressure.

MXenes have been hybridized with several inorganic materials, such as transition metal oxides, phosphides, nitrides, and chalcogenides, using hydrothermal/solvothermal synthesis [87]. MXene-based nanocomposites produced through this method have demonstrated potential in energy storage applications, particularly as supercapacitors, due to their exceptional electrochemical energy storage efficiency and cycle life. Furthermore, the solvothermal technique has been used to integrate MXenes into other material matrices, such as SnTe, to improve their electrical and thermal transport properties, opening doors for high-performance thermoelectric materials [88]. Researchers synthesized a nanohybrid, Ti_3_C_2_T_x_ (MXene)/CoS_2_/CuCo_2_S_4_, through hydrothermal synthesis for application in the design of supercapacitor devices. The process involved coating CuCo_2_S_4_ particles and sheet-like CoS_2_ onto Ti_3_C_2_T_x_ nanosheets. By adjusting the ratio of MXene to CoS_2_/CuCo_2_S_4_, the electrochemical efficiency of the hybrid nanocomposite electrode was significantly enhanced [89]. By adjusting the reaction temperature during hydrothermal synthesis, precise control of the lateral particle size of MQDs can be achieved, and large-scale production can be achieved. Further research is necessary to optimize hydrothermal parameters and develop new synthetic methods to overcome MQD oxidation while maintaining structural integrity for practical applications. Ti_3_C_2_T_x_ quantum dots (MQDs) can be synthesized from Ti_3_C_2_T_x_ MXene nanosheets using the solvothermal method. For the synthesis of MQDs with various sizes and well-dispersed quantum yields, different organic solvents, such as ethanol, DMSO, and DMF, can be used. The polarity, boiling point, and degree of oxidation of the solvent may influence the characteristics of the MQDs. Solvothermal synthesis can be used to precisely control the morphology of MQDs and create stable and evenly distributed samples that have numerous possible applications [86].

### 4.2. Deposition Methods

A variety of deposition methods, including electrodeposition, atomic layer deposition (ALD), photodeposition, and CVD, are applied to synthesize MXene composites. These methods provide precise control of the deposition process, enabling the production of customized materials with cutting-edge characteristics [85]. Large, high-quality 2D materials, electronic components, and flexible optoelectronics are all readily and efficiently made using the CVD technique. A wide range of materials, including semiconductors, nanocomposites, alloys, and metals, can be manufactured using this technique. [90]. ALD, similar to CVD, divides reactions into two different halves and ensures that the precursor materials do not interact while being deposited. By precisely controlling film development at the atomic level, this technique enables the deposition of thin films that exhibit excellent conformality (uniform and precise adhesion to the substrate’s contours, ensuring smooth and uniform coating) and consistent thickness on complex substrates. ALD films have been recognized for their superior homogeneity, chemical bonding to the substrate, and lack of pinholes [91].

MXenes are combined with other transition metal oxides, phosphides, C-based materials, and metals to produce MXene hybrids by electrodeposition techniques. By applying an electric field, this approach uses MXene and other compounds to deposit them onto electrodes or substrates. This method enables the creation of MXene composites with the required characteristics by allowing user control of the composition, shape, and thickness of the deposited layers. Metallic MPs are deposited onto MXene surfaces by a technique known as photodeposition, which utilizes light irradiation. This simple and controlled technique allows the integration of MXenes with metallic components and the selective deposition of NPs [92,93]. Overall, the deposition methods offer very accurate control and customization of MXene composites, but there are certain challenges that must be addressed, including high production costs, oxidation protection, and scalability. Despite these challenges and setbacks, MXene composites are still a valuable resource for creating high-quality materials suitable for various applications and can be designed with this approach with proper optimization.

### 4.3. Solution Processing

This method is often used to create polymer composites of MXenes. Due to their hydrophilic nature and functional groups, MXene nanosheets are extremely compatible with polar solvents, including N,N-dimethylformamide (DMF), water, and dimethyl sulfoxide (DMSO) [94]. This method disperses premodified MXenes and polymer components in a variety of solvents to increase the dispersibility of the NPs. This approach results in easier combinations of various polymers, such as cellulose, polyethylene oxide, chitosan, polyvinyl alcohol, and polyacrylate, with MXenes. Inorganic compounds such as transition metal oxides and chalcogenides can also be hybridized with MXenes through the use of solution processing techniques [95,96]. Using processing techniques to create MXene composites has produced a number of benefits despite a handful of possible downsides. Small-scale manufacturing in laboratories is made possible by how easily MXenes can be incorporated into polymers with proper optimization of the solvent removal process after blending. The flexibility of solution processing in utilizing different ingredients and solvents allows for the development of MXene composites with a wide variety of highly beneficial attributes.

In addition to developing composites with substandard mechanical properties, the method itself may produce a sizable quantity of environmental waste. The scaling and practicality of solution processing may also be hampered by the difficulty of removing solvents after the blending process [85]. The selection of solvents and surface modifications can also enhance the stability of MXenes and expand the range of compatible polymers. Additionally, solution-based processing techniques, such as epoxy, polysulfone, PVDF, polyacrylonitrile, and polycaprolactone, have been utilized for various polymer matrices, highlighting the versatility and potential of this method for creating MXene composites [97].

### 4.4. Dropcasting and Adsorption

Dropcasting and adsorption are two nonreactive techniques used to synthesize MXene-based composites. These methods have the advantage of avoiding high-temperature treatment since they rely on electrostatic and van der Waals forces for self-assembly [98,99]. MXene/C-based hybrids are usually synthesized by the adsorption technique. By drop-casting a colloidal solution of MXene onto a slot antenna array, an ultrathin film may be created [100]. This approach allows quick processing, high uniformity, and effective loading onto substrates. In general, drop casting and adsorption techniques offer straightforward and effective solutions for producing MXene-based composites without requiring high-temperature processing. This approach also offers flexibility in incorporating different secondary materials.

**Table 1:** Fabrication of MXene conjugates and their application.

Types of Conjugates		Method of Preparation/Design	Reason for Usage	Application	Reference
Metal conjugates	Pt-TBA−Ti_3_C_2_T_x_ (Platinum nanoparticle deposited MXene)	Atomic layer deposition (ALD) Etching agent: HF Delaminating agent: TBAOH	Excellent hydrogen evolution reaction (HER) activity, Stability, homogenous dispersion Excellent conductivity	Effective commercial catalysts for HER.	[91]
	Ti_3_C_2_/ZIF-67/CNTs	In situ synthesis with mixing	Amplified detection signal High stability and reproducibility	Electrochemical sensors in pharmacology	[101]
Quantum dots nanoconjugates	Nonoxidized MQDs-Ti_3_C_2_T_x_	Self-designed micro explosion method	Destruction of tumor blood vessel integrity and cancer cell death Excellent biocompatibility with normal cells In vivo therapeutic performance shows highly desired tumor suppression and killing effect	Cancer Catalytic Therapy	[102]
	PLL-protected Ti_3_C_2_ MQDs	Sonication cutting Hydrothermal synthesis	Excellent blue luminescence High sensitive fluorescence for detection of trypsin and Cyt-c	Fluorometric determination of cytochrome c and trypsin	[103]
MXene-Polymer nanoconjugates	Ti_3_C_2_ functionalized with soybean phospholipid (SP), and poly (lactic-co-glycolic acid) (PLGA)	Two-step exfoliation approach of HF etching then TPAOH intercalation	Highly effective photothermal therapy (PTT) agent for tumor therapy Novel photothermal agent used for cancer therapy	Anticancer therapy	[104]
	MnO_x_/Ti_3_C_2_–SP	Simple redox reaction	Multifunctional theragnostic agent for MR/PA imaging-guided PTT against cancer In vivo biocompatibility evaluation ensures safe clinical translation	pH-Responsive MRI-Guided Tumor Hyperthermia	[105]
MXene hydrogels	MXene Composite Hydrogels	HF etching Liquid-phase exfoliation (LPE)	Dual-modality PTT and chemotherapy against cancer Favorable biocompatibility	Light-Induced Swelling and Bimodal Photothermal Chemotherapy	[79]
	Ti_3_C_2_/PAM NC hydrogels	In situ free radical polymerization	Excellent mechanical properties, high deformability, and stretching ability due to uniform fine structures High drug load and drug release percentages, making them suitable for biomedicine applications	Enhanced mechanical and drug release properties	[106]
Biomimetic MXene	Nb_2_C plasmon	In situ preparation	Effective in destroying tumor cells due to lower heat resistance compared to normal cells Photoacoustic imaging revealed increased blood perfusion at the tumor site	Targeted cancer treatment	[107]
	MXene and AuNP@BLM (biomimetic bilayer lipid membrane)	Drop-casting method	Specificity shown by discriminating noncomplementary DNA and mismatched sequences Possibility of microfluidic platform assembly for BRCA1 diagnosis	Bilayer lipid membrane biosensor for zeptomole detection of BRCA1 gene	[108]
	MoS_2_ QDs-MXene heterostructure and Au NPs@biomimetic lipid layer	Microwave-assisted hydrothermal method	Clinical detection of miRNA-135b in exosomes in the ascites of gastric cancer patients Application as a potential diagnostic tool for gastric cancer	ECL sensor in cancer detection	[109]
MXene-graphene	Au-Pd/MXene/LSG (Laser-scribed graphene)	Drop-casting	It is a cost-effective electrode material and biocompatible High electrocatalytic activities towards the oxidation of ascorbic acid, dopamine, and uric acid Skin-adaptable, user-friendly, and label-free direct measurement	Detection of ascorbic acid, dopamine, and uric acid	[110]
	TiC_2_T_x_ nanolinks and graphene	Liquid-phase exfoliation HF etching Delamination	Integrated temperature sensing with strain sensors allows accurate strain measurement without complex temperature compensation Superior sensitivity in strain and temperature measurements	All-printed thin-film bimodal sensor	[111]

## 5. MXene-Based Conjugates for Nanotheranostics

Nanocomposites are gaining popularity due to their unique ability to integrate two or more species. Two-dimensional MXenes have gained widespread attention from scientists owing to their distinctive structural properties, metallic conductivity, diverse surface chemistry, large surface area, hydrophilicity, biocompatibility, and customizable particle size. These characteristics make them useful for generating multifunctional nanocomposites. One such advanced application of nanocomposites is the preparation of nanotheranostic platforms that integrate diagnostic and therapeutic functions into a single nanoscale platform. Photothermal treatment, which involves near-infrared (NIR) laser irradiation between 700 and 1300 nm, is a less intrusive approach to cancer treatment than standard approaches such as chemotherapy and radiotherapy. Photothermal agents play an important role in optimizing PTT and must meet certain requirements, such as an excellent extinction coefficient (α) for efficient laser absorption, exceptional photothermal conversion efficiency (PCE) (η) to convert light into heat, and excellent photothermal stability. MXenes, particularly Ti_3_C_2_, have demonstrated outstanding light absorption and inherent efficiency (nearly 100%), outperforming other photothermal nanomaterials, such as CNTs [112]. The localized surface plasmon resonance (LSPR) effect caused by the semimetal characteristics of MXenes accounts for their outstanding light absorption and conversion capacity [104]. However, the practical application of MXenes is limited by their sensitivity to oxidative breakdown, which has a specific influence on their prospective use in biological situations. This problem may be solved by conjugating the MXene with the proper components.

### 5.1. MXene–Polymer Conjugates

MXenes suffer from limitations in biomedical applications despite their substantial features, such as a high surface area, improved hydrophilicity, reduced toxicity, conductivity, and size tunability. These limitations include reduced stability in physiological settings, insufficient sustained and regulated active release, and limited biodegradability. MXene–polymer nanocomposites have evolved as a solution to these problems. Lin and coworkers fabricated ultrathin Ti_3_C_2_ MXene nanosheets of atomic thickness for efficient photothermal destruction of tumor cells [104]. The nanosheets were exfoliated via exfoliation via HF etching followed by tetrapropylammonium hydroxide (TPAOH) intercalation. The LSPR effect exhibited by the Ti_3_C_2_ nanosheets resulted in a good PCE of 30.6% at 808 nm. The physiological stability of Ti_3_C_2_ was enhanced by modifying it with soybean phospholipid and PLGA. In vivo studies revealed efficient 808 nm NIR-induced cancer ablation by intratumoral injection of Ti_3_C_2_-SP nanosheets (20 mg/kg) or by 2 mg/kg Ti_3_C_2_-PLGA nanosheet implants. Significantly, the phase transformation features of the PLGA/Ti_3_C_2_-SP implant enable cancer cell death while also ensuring that no implanted component leaks into the circulation, resulting in great in vivo biosafety. Szuplewska et al. described the localization of Ti_2_C-PEG nanoflakes in cancerous cells and suggested that MXene has different affinities for membranes of normal and cancerous cells, which may be related to its selectivity and nanosized planar configuration [113].

**Figure 3:**
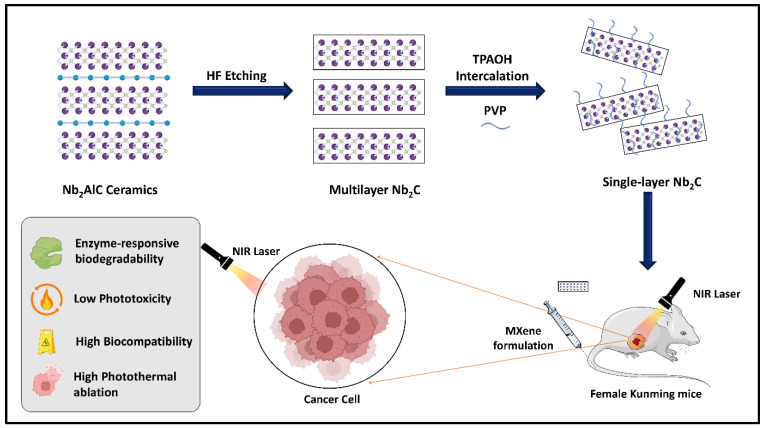
Diagrammatic illustration of the preparation of 2D biodegradable Nb_2_C modified with PVP for in vivo photothermal tumor ablation.

Feng et al. constructed Mo_2_C MXenes for photothermal tumor hyperthermia for the first time [114]. The exfoliated Mo_2_C was functionalized with polyvinyl alcohol (PVA) to form Mo_2_C-PVA nanoflakes, which degraded faster and exhibited improved biocompatibility. The as-prepared Mo_2_C-PVA nanoflakes displayed broad absorption spectra in both the NIR I and II regions, with better PCEs of 24.5% and 43.3%, respectively. The photothermal cancer ablation potential of Nb_2_C-PVP in the NIR I and II regions was explained by Lin and coworkers [115]. The PCE of the prepared nanocomposites was 36.5% and 46.5% for the NIR I and II regions, respectively. The PVP coating (20.36%) on the Nb_2_C nanosheets confirmed the efficient intratumoral localization of the nanocomposite, with a systemic circulation half-time of 1.31 h. PT ablation at 808 nm and 66 °C was observed at a temperature of 61 °C under a 1064 nm NIR laser (Figure 3). Its biosafety and biocompatibility were demonstrated by its rapid excretion and absence of phototoxicity. Enzyme-activated biodegradation of the Nb_2_C-PVP nanocomposite was achieved using human myeloperoxidase (hMPO) and hydrogen peroxide. Liu and coworkers developed a Ti_3_C_2_ MXene-based nanocomposite for synergistic phototherapy against cancer [78]. The exfoliated ultrathin MXene exhibited an exceptional extinction coefficient (28.6 Lg^−1^·cm^−1^) and a good PCE of approximately 58.3% upon 808 nm NIR irradiation. The nanosheets were endowed with multiple functions through layer-by-layer surface functionalization with doxorubicin (DOX) and hyaluronic acid (HA). The prepared nanocomposite system exhibited an enhanced permeability and retention (EPR) effect and precise targeting of CD44+ T cells, leading to improved tumor accumulation at a low dose.

The presence of functional groups on the MXene surface enables polymer functionalization. As a result, MXene–polymer nanocomposites have a number of favorable properties. These include excellent photothermal conversion efficiency, selectivity, and stimulus reactivity, particularly when targeting cancer cells. Furthermore, these nanocomposites have improved electron sensitivity, antimicrobial capabilities, and a variety of other advantageous qualities. The resultant nanocomposites offer significant potential for enhancing biological applications by combining the unique features of MXenes with the adaptability of polymers.

### 5.2. MXene–Metal Conjugates

MXene-based metal-conjugated hybrids are created by integrating metallic nanocomponents such as Ag, Au, Pd, and Pt onto the surface of MXenes, increasing the stability and activity of the nanocomposite and enhancing its suitability for biomedical applications. Compared with bulk materials, metallic NPs offer superior magnetic, electrical, and optical characteristics, making them interesting diagnostic agents in MXene–metal nanocomposite fabrication. Several limitations of traditional chemotherapy can be solved by combining surface-modified MXenes with metal NPs. In a recent study by Xi and coworkers, Ti_3_C_2_Cl_2_ MXene-based nanocomposites were developed via an HF-free technique [116]. The Au/Pt/Ti_3_C_2_Cl_2_ nanocomposite was developed by a single-step self-reduction approach, where MXene can act as an efficient reducing agent. Because of their large specific surface area and characteristic accordion arrangement, the MXene nanoflakes acted as both a reducing agent and a supporter during the reduction process. The as-prepared nanocomposite demonstrated outstanding catalytic activity in H_2_O_2_-TMB and O_2_-TMB systems, as well as low cytotoxicity and acceptable biocompatibility. The biosensor enabled in situ sensing of H_2_O_2_ produced by HeLa cancer cells along with GSH sensing. Compared to other previously published approaches, the colorimetric biosensor created in this research has a lower detection limit and a wider detection range, indicating its possible utility for future development. Furthermore, the biosensor’s sensitivity and repeatability for colorimetric detection of H_2_O_2_ were assessed. Liu et al. synthesized an MXene@Au-PEG nanoplatform for improved loading of DOX with pH-triggered and NIR light-induced drug release [117].

**Figure 4:**
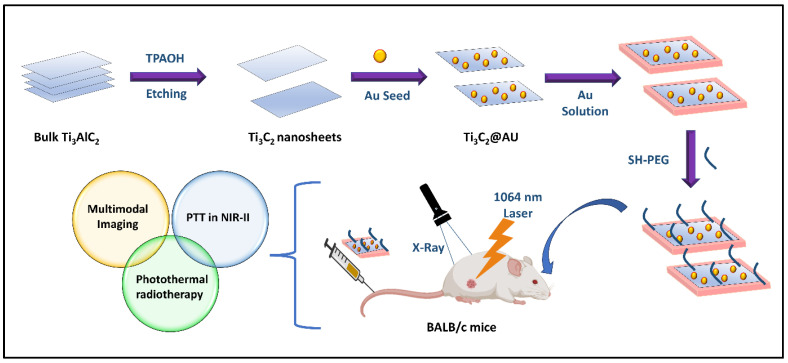
Pictorial representation of Ti_3_C_2_@Au synthesis, PEGylation, and in vivo PA/CT dual-modal imaging-guided photothermal therapy in combination with radiotherapy.

The exfoliated MXene nanosheets were functionalized with PEG aldehyde chains via Au NPs, forming an MXene@Au-PEG-DOX nanocomposite that exhibited efficient photothermal stability and biocompatibility both in vitro and in vivo. Furthermore, the composite demonstrated synergistic photo/chemotherapy for cancer therapy due to the good photothermal conversion capacity of both the Au and MXene particles. Tang and coworkers fabricated Ti_2_C_3_@Au nanocomposites via a seed growth approach for image-guided tumor treatment [118]. The exfoliated nanosheets were modified with poly(allylamine hydrochloride) to form Ti_3_C_2_-PAHs with a change in zeta potential from −24 mV to 40 mV, which assisted in the negative charge of the Au particles via electrostatic interactions. The growth of Au particles on the surface of the Ti_3_C_2_ nanosheets occurred via electrostatic interactions between the Ti_3_C_2_ nanosheets and negatively charged Au seeds. The biosafety and compatibility of the Ti_3_C_2_@Au composite could be efficiently enhanced by the addition of thiol functional groups. Furthermore, this approach greatly improved the optical absorbance in the NIR biological window. The excellent X-ray attenuation capability and better optical efficiency of the nanocomposite enabled dual-modal imaging (Figure 4). Compared with PVP-modified Ti_3_C_2_ nanosheets, the PEGylated Au composite of MXene improved tumor cell killing, which indicated that the Au-MXene composite had a greater PCE. The in vivo PTT efficacy and radiotherapy efficacy of the nanocomposite were assessed in 4T1 tumor-bearing BALB/c mice. As predicted, the PTT + radiotherapy combination group demonstrated the most severe tumor cell destruction, while the other groups showed minimal or no cancer cell destruction.

For PTT applications, MXenes cannot be used to accurately diagnose and treat the effective killing of tumor cells. To address this issue, An et al. developed an “all-in-one” nanoconjugate of Ti_3_C_2_ MXene nanosheets for MRI-guided hyperthermia and chemotherapeutic tumor destruction [119]. This was accomplished by bonding manganese (Mn) ions on the Ti_3_C_2_ nanosheet surface, followed by functionalization with biocompatible PEG to form PEG@Ti_3_C_2_-Mn. As the Mn particles exhibit Fenton-like catalytic characteristics (the ability of a material to catalyze the generation of highly reactive hydroxyl radicals (·OH) from hydrogen peroxide (H_2_O_2_)) along with magnetic properties, the prepared nanocomposite could act as a chemodynamic agent by converting H_2_O_2_ to OH radicals and MRI contrast agents. Although prior research has revealed the efficacy of cancer cell killing, only a few studies have integrated different therapeutic modalities to produce superior outcomes. Furthermore, as developing inorganic nanomaterials, MXene and MnO_2_ nanozymes have excellent size fits. As a result, to improve the efficacy of multimodal tumor ablation, Li et al. developed a simple and fast process for preparing MnO_2_-Ti_3_C_2_ nanocomposites and evaluating their photothermal potential [120].

### 5.3. MXene–Graphene Conjugates

MXene nanosheets have demonstrated high efficiency as a hybridization matrix compared to graphene. Various MXene–graphene hybrid composites exhibit exceptional structural robustness, conductivity, and flexibility qualities, as well as unique electrical, electrochemical, and mechanical features. When used in a PEG matrix, these composites demonstrated increased through-plane heat conductivity. The improved electromagnetic interference (EMI) shielding performance of the developed composites reached 36 dB at a thickness of 2.5 mm. Magnetic MXene (Ti_3_C_2_T_x_)-reduced graphene oxide aerogels with magnetic nickel nanochains were shown to have appropriate multifunctionality, hydrophobicity, and heat insulation activity. The 2D materials, such as macroscopic hydrogels, continue to be very fascinating for constructing 3D structures. Wychowaniec and colleagues reported a unique method for producing chemical intersheet crosslinks between Ti_3_C_2_T_x_ and graphene oxide via an ethylenediamine-mediated process to produce an rGO-MXene hydrogel [121]. They introduced a temperature-sensitive crosslinking technique resulting in the formation of hydrogels with characteristic chemical and 3D architectural properties. The first synthesized hydrogel composite demonstrated an improved hydrophobic surface with a stiffer surface and an elastic modulus of approximately 40 kPa. Fluorescent confocal microscopy analysis of three human cell lines, namely, SH-SY5Y, MSU 1.1, and HeLa, demonstrated that these cells created an extended 3D cellular network on these hydrogel composites. After two days of incubation with the rGO-MXene hydrogel composite, the neuroblastoma cell line SH-SY5Y self-assembled into neurosphere structures, which further disassembled into diffusive cellular networks on the seventh day of incubation. The rGO-based hydrogel structures facilitated cell migration and allowed nutrients to diffuse throughout the scaffolds. Prolonged production of cells with migration-driven cytoskeletal characteristics, such as filopodia, neurites, and lamellipodia, demonstrated strong interpore penetration capacities in all cultivated cell types. This behavior seems to be more pronounced in the rGO-MXene hydrogel composite than in the rGO hydrogels themselves, resulting in unique cellular interactions with the Ti_3_C_2_T_x_ flakes, which are cell-type independent. These findings point to the distinct biological activities of these materials and their potential application in the realms of tissue engineering and customized medicine. A unique 3D composite aerogel (3D patent blue (PB)/rGO/MXene composite aerogel (GMA)) was created by Zhao and colleagues and is used to sense the secretion of H_2_O_2_ from live cells in real time [122]. The excellent peroxidase-like activity and clear porous structure of the 3D composite aerogel were demonstrated. The outstanding electrocatalytic activity towards H_2_O_2_ was demonstrated by an electrochemical sensor based on 3D PB/GMA. It could successfully separate cancer cell lines from normal cell lines when used for real-time tracking of H_2_O_2_ release from live cells, suggesting that it has significant potential for use in clinical diagnostics. In another study by Zhao et al., a biosensor of a ZnO nanorod sensor was fabricated with an rGO-MXene nanocomposite for GSH sensing [123]. It was found that covering ZnO nanorods with RGO/MXene-derived TiO_2_ could effectively promote ZnO nanorod development and greatly enhance the performance of ZnO nanorod-based GSH sensors.

### 5.4. MXene–Hydrogel Conjugates

**Figure 5:**
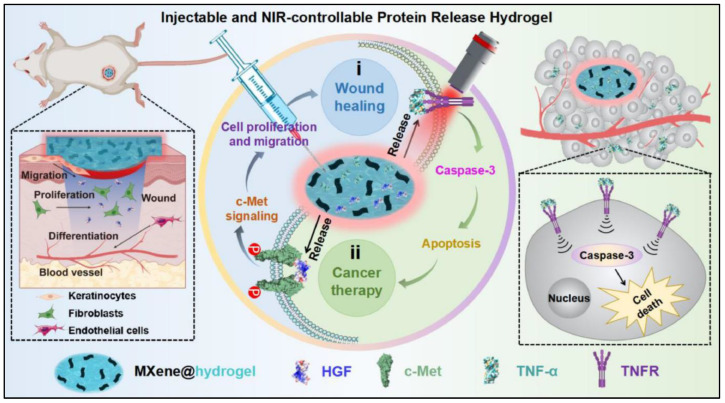
Schematic illustration of the potential of MXene–hydrogel conjugates for cancer treatment. Reproduced with permission from [125].

The conjugation of MXenes with hydrogels is highly important due to their critical function in the treatment of cancer. Hydrogels, which are recognized for their ability to hydrate substances, high loading capacity, and controlled release characteristics, can be used to effectively transport and deliver various molecules to specific target sites. By regulating the clearance and denaturation of proteins and other therapeutic moieties in challenging physiological environments, this integration effectively preserves localized concentrations at disease sites while reducing the occurrence of adverse effects associated with burst or off-target release [124]. Despite this, hydrogels encounter numerous obstacles due to the limited spatial–temporal controllability exhibited by diffusive triggering molecules. These issues can be addressed by having an exogenous control or trigger (such as ultrasound, magnetic fields, or light) to monitor and regulate the release of such molecules. With this concept of employing a hydrogel–MXene conjugate, a study [125] developed an injectable composite hydrogel system that responds to near-infrared (NIR) light (Figure 5). This was achieved by combining Ti_3_C_2_ MXene and protein therapeutics with an agarose hydrogel, which enabled the controlled release of functional proteins in response to specific cellular signals upon exposure to NIR light. The platform’s adaptability was demonstrated through the development of a composite hydrogel system (MXene@agarose/TNF-α) that incorporates tumor necrosis factor-α (TNF-α) and enables the use of NIR light to modulate proapoptotic signaling in tumor spheroids in vitro. By utilizing the strong ability of the NIR laser to diffuse deep into tumors, MXene@agarose/TNF-α enabled the effective elimination of tumors from a xenograft animal model. The MXene@hydrogel/protein system allowed multichannel modification of the release rate as well as precise “on/off” controlled drug release.

Stimulus-responsive DNA hydrogels, notable for their biocompatible three-dimensional network architectures, show great potential as options for cancer therapy. In the context of exploring the potential of MXene-hydrogel conjugates for cancer treatment, another study [126] developed a robust framework by combining Ti_3_C_2_T_X_-based MXenes with DNA hydrogels, resulting in a highly effective synergistic photothermal–chemical cancer treatment system. When exposed to NIR, the MXene material generated heat, which led to a reversible change from a gel to a solution in the MXene-DNA hydrogel loaded with DOX. This approach enables precise and controlled delivery of the medicine for targeted cancer treatment. After the NIR irradiation stopped, the system returned to its initial state, demonstrating its ability to adapt and efficiently achieve targeted cancer treatment with minimal adverse effects. After surgery, residual tumors are highly likely to recur; thus, techniques for eliminating residual tumors must be developed. Antitumor drugs administered locally after surgery have been demonstrated to be effective at eradicating surviving tumor cells. Nevertheless, there are still issues with using these drugs to treat patients consistently and effectively over the long term. Focusing on this issue, Wang et al. [127] introduced an innovative strategy by developing a photothermal-responsive biopaster created by systematically arranging propyl gallate (PG)-grafted MXenes onto a calcium-alginate hydrogel. This biopaster aimed to inhibit tumor recurrence after surgery. The organized configuration of the MXene nanosheets with PG grafting exhibited an extended degradation period in comparison to that without PG grafting, resulting in a consistent photothermal response that persisted for 14 days. As a result, the biopaster that was created showed a long-lasting ability to use heat from light to treat diseases, effectively eliminating any remaining tumors and preventing tumor recurrence after surgery. To summarize, MXene–hydrogel conjugates offer a promising approach for precise cancer treatment. The studies demonstrated the controlled drug release, photothermal sensitivity, and ability of this conjugate to minimize tumor recurrence, highlighting its potential against cancer while minimizing adverse effects.

### 5.5. Biomimetic MXenes

**Figure 6:**
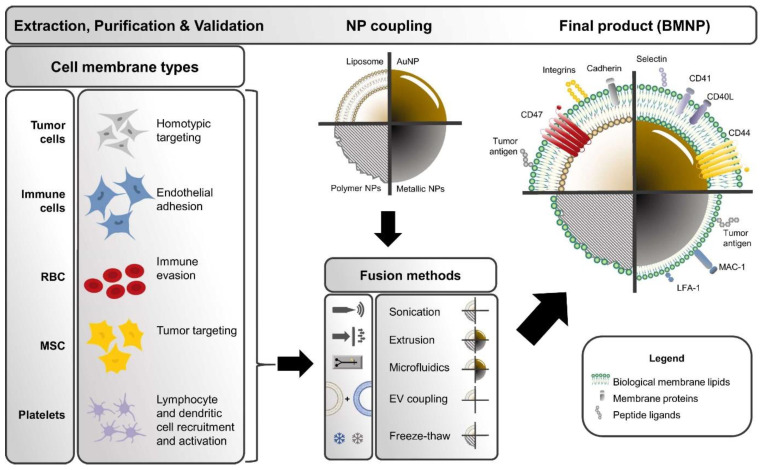
Overview of cell membrane coating on NPs. Reproduced with permission from [129].

Recent progress in enhancing the biocompatibility of NPs has involved coating or fusing NPs with cell membranes (Figure 6). This endows NPs with exceptional biocompatibility and enhances their tumor targeting ability and circulation time [128,129]. By integrating diverse immune cell membranes, including bacteria, erythrocytes, platelets, and white blood cells, the physicochemical characteristics of core NPs synergize with the biological attributes of native source cells. Such immune-cell engineering is evolving as a promising research area with potential applications in nanoscale biomedicine to overcome the limitations of core NPs. In this context, the nature-inspired modification of MXenes is nascent, yet few reports utilize this concept.

Photonic crystal arrays were fabricated in one such study [130], inspired by the wettability and adhesion of the Stenocara beetle. The fluorescence resonance energy transfer mechanism was employed to successfully inhibit the fluorescence signals of DNA probes modified with quantum dots using MXene nanosheets, even in the absence of specific targets. In addition, the development of nanosheets led to an improvement in the contrast of the structural colors. The bioinspired MXene films derived from Ti_3_C_2_T_x_ exhibited exceptional flexibility. The MXene family has been identified as a very effective group of near-infrared II (NIR-II) photothermal agents. These agents are distinguished by their significant specific surface area and impressive ability to convert light into heat. One study [131] explored the ability of MXenes to combine PTT with immunotherapy. The developed Nb_2_C nanosheets demonstrated a significant PCE upon irradiation with a 1064 nm laser. The application of NIR light was found to improve tissue penetration. The stability and loading efficiency of the nanosystem were enhanced by coating with polydopamine, and to further address the issue of excessive blood clearance, a red blood cell layer coating was used. The efficacy of the created Nb_2_C@PDA@RBC nanoplatform was observed through primary tumor suppression and inhibition of secondary tumor development. This effect was primarily ascribed to the enhanced immune response, and overall, the results demonstrated that the Nb_2_C@PDA@RBC NPs exhibit potential as promising nanoplatforms for effective photothermal therapy and immune therapy in the treatment of tumors.

**Figure 7:**
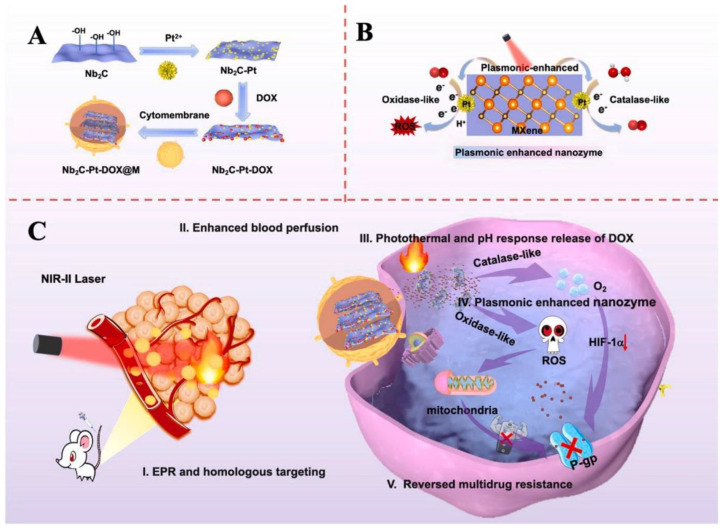
Pictorial illustration of (**A**) the preparation of biomimetic plasmonic assemblies, (**B**) catalytic activity, and (**C**) therapeutic hypothesis. Reproduced with permission from [107].

Another study [107] presented an innovative approach for the treatment of tumor cells by utilizing a biomimetic plasmonic assembly that emulates the physiological mechanisms of the human body. The assembly consisted of a plasmon core made of Nb_2_C, which was coated with a cancer cell membrane. This membrane coating incorporates Pt nanozymes and DOX. The catalytic nature of Pt nanozymes in producing O_2_ and ROS can be enhanced by the generation of hot electrons from the Nb_2_C plasmon when exposed to NIR-II laser light. These compounds can mitigate tumor hypoxia, suppress P-glycoprotein (P-gp) expression, and synergistically interact with DOX to counteract multidrug resistance and ultimately enhance tumor therapy efficacy (Figure 7). A novel biosensor intended for biosensing BRCA1 gene mutations in breast cancer patients was described in another study [108]. In addition to gold NPs, biosensors integrate biomimetic bilayer lipid membranes (BLMs) with MXene nanosheets. The electrochemical activity of the designed biosensor was illustrated through the attachment of a DNA probe to the BLM/MXene surface, followed by hybridization with the DNA target. The biosensor exhibited remarkable specificity, reproducibility, and sensitivity for the BRCA1 gene, along with a low sensing limit. The authors claim that the remarkable performance of their biosensor can be ascribed to the unique attributes of MXene and BLM. Recent findings have integrated the biomimetic characteristics of MXenes for diverse applications, such as the detection of miRNA-135b [132], cuproptosis-based immunotherapy [133], and the design of cascaded-enzyme nanoreactors [134]. To summarize, the use of biomimetic approaches to alter MXenes holds great potential for various nanoscale biomedical applications. With continued research efforts, it is very likely that we will see additional ground-breaking uses of this technology in the near future.

### 5.6. MXene–Quantum Dot Conjugates

**Figure 8:**
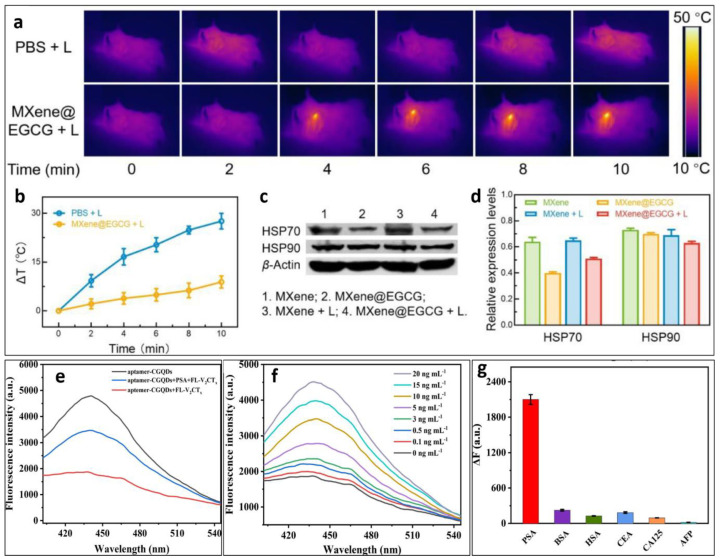
Applications of MXene-QD conjugates. (**a**) Photothermal images of 4T1 tumor-bearing BALB/c mice administered MXene@EGCG (illumination: 808 nm laser) and (**b**) the temperature increase curves of the tumors. (**c**) Western blots showing HSP70 and HSP90 expression in tumor cells from mice. (**d**) Relative expression of HSPs. Fluorescence emission spectra of (**e**) aptamer-CGQDs with and without FL-V_2_CT_x_, (**f**) the aptasensor with the addition of PSA, and (**g**) the ΔF of the aptasensor for PSA, CEA, CA125, AFP, BSA, and HSA. (**a**–**d**) Adapted with permission from [136] and (**e**–**g**) adapted with permission from [137].

Conjugating quantum dots is crucial in the field of cancer theranostics because they efficiently combine both diagnostic and therapeutic abilities [135]. When combined with cancer-specific biomarkers, these QDs possess distinct optical properties that allow them to display remarkable sensitivity and specificity in detecting tumor cells. Furthermore, the ability of these entities to effortlessly integrate with other imaging methods, including fluorescence, magnetic resonance imaging (MRI), and photoacoustic imaging, enables comprehensive assessments of tumor characteristics. The application of quantum dot fluorescence enables real-time monitoring of therapeutic responses, allowing timely modifications of treatment regimens. In one such study [136], the authors utilized the properties of Ti_3_C_2_T_x_ MXenes, such as the PCE, cargo loading and ability to scavenge free radicals. The common limitation associated with PTT and hyperthermia is heat shock protein (HSP) overexpression by impaired malignant cells. These HSPs tend to protect and repair damaged proteins and thus reduce therapeutic efficacy. To address this issue, the authors developed metal-polyphenol nanodot-coated nanosheets. The compound epigallocatechin gallate (EGCG) was chosen as the polyphenol for the development of metal-polyphenol nanodots. EGCG is known for its capacity to decrease HSP70/90 expression in tumor cells, and it also has anti-inflammatory properties that ultimately enhance the effectiveness of PTT against cancer. These nanosheets, which measure approximately 240 nm in size, were optimized for accumulation in tumors by employing optimal etching, intercalation, and ultrasonication techniques. The purpose of this optimization was to take advantage of the enhanced EPR effect, which promotes therapeutic agent accumulation in tumor tissues. The nanodots were generated by combining Fe^3+^ ions with EGCG through metal-polyphenol interactions. When irradiated at 808 nm, the MXene@EGCG composite demonstrated a significant PCE of 29.2%. Furthermore, when irradiated, this composite revealed the potential to considerably increase the temperature of the tumor. The formulation also suppressed HSP70 expression both in vitro and in vivo (Figure 8a–d). Furthermore, the combination of MXene@EGCG showed great efficacy in decreasing the inflammation generated by PTT both in vitro and in vivo while causing little harm to animals. These data demonstrated that MXene@EGCG could be a promising cancer therapeutic candidate. The MXene-QD conjugate has also proven important in cancer biosensing.

For instance, one study [137] employed an MXene-QD conjugate as a fluorescent aptasensor to detect the prostate-specific antigen (PSA). Upon the conjugation of graphene QDs with few-layer vanadium carbide nanosheets, the fluorescence intensity of the QDs decreased in proximity to that of PSA, with good specificity and a sensing limit of 0.03 ng/mL (Figure 8e–g). The developed aptasensor also demonstrated successful biosensing of PSA in serum obtained from patients with prostate cancer. These findings emphasize the potential of the aptasensor for clinical application. Several other studies have reported on its application in the sensing of different biomolecules, especially RNA, such as miRNA-135b [132], miRNA-221 [138], and miRNA-421 [139]. Overall, complete exploration of the potential of MXene-QD conjugates has not yet been achieved, leaving open the prospect of conducting clinical trials.

### 5.7. MXene–Radio Conjugates

Radiotherapy involves the application of ionizing radiation (internally or externally) to restrict abnormal tumor cell growth and to inhibit tumor relapse after surgical removal [140]. In the context of nanoconstructs, radioisotopes are conjugated to achieve either therapeutic effects (^177^Lu, ^67^Cu, ^186^Re, ^90^Y, ^111^Ag) or diagnostic abilities (^99m^Tc, ^131^I, ^67^Ga, ^111^In, ^18^F). The preparation of precise radionuclide delivery platforms is crucial in the field of internal radiation therapy, with the objective of optimizing tumor destruction while minimizing adverse effects [141]. These platforms may be employed in conjunction with other therapeutic approaches to achieve synergistic antitumor activity. The US FDA has approved many radiopharmaceuticals for cancer treatment [142]. Due to their unique properties, MXene materials hold great promise for both internal and external radiotherapy applications. Internally, MXenes can be utilized as efficient carriers for radioisotopes, which can facilitate targeted internal radiation therapy. Externally, the high X-ray attenuation capability of MXenes makes them potential candidates for enhancing the efficacy of external beam radiotherapy [143]. This approach could improve imaging and treatment outcomes in cancer therapy. In light of this, a study reported [118] the synthesis of Ti_3_C_2_ nanosheets using a seed growth technique. The addition of gold to the Ti_3_C_2_ nanosheets increased their optical NIR absorption, which was crucial for effective radiotherapy. The resulting nanocomposites were effective for photoacoustic and computed tomography dual-modal imaging, and their mild photothermal activity helped to improve tumor oxygenation, greatly increasing the efficacy of radiotherapy. Importantly, the administered dose did not cause any discernible long-term toxicity, demonstrating the potential of MXene-based multifunctional nanocomposites for external radiotherapy.

MXenes are emerging as promising contenders for biomedical applications, particularly in diagnostic modalities such as positron emission tomography (PET), computed tomography (CT), and magnetic resonance imaging (MRI). However, their intrinsic diamagnetic nature limits their bioimaging applications. To overcome this limitation, a covalent functionalization approach was employed in a previous study [144] in which the chelating agent diethylenetriaminepentaacetic acid (DTPA) was incorporated into MXenes, followed by complexation with Gd^3+^ ions. This approach imparted paramagnetic characteristics that made the MXenes conducive to T1-MR imaging. The covalent approach also enhanced MXene stability, mitigated self-aggregation, and facilitated a high degree of PCE, demonstrating potential applications in photothermal therapy. This work not only introduces an MRI contrast mechanism but also advances covalent functionalization approaches for MXenes, expanding their bioapplication potential. This study further revealed the apparent concentration-dependent magnetic relaxation time of MXene flakes, enabling spatially resolved flake distribution estimation, surface protection against oxidation and increased cytocompatibility in physiological environments. The chelation of Gd^3+^ ions in this covalent approach proves superior to electrostatic chemisorption, revealing a versatile and robust avenue for MXene bioapplications. MXenes offer a promising avenue for radiolabeling, which can be achieved either directly or through the utilization of chelating agents. The versatility of surface modification techniques available for MXenes opens up numerous possibilities for incorporating radioisotopes, presenting a compelling opportunity for enhancing internal radiotherapeutic applications. Despite this potential, a noticeable research gap exists, as there are currently no studies examining the ability of MXenes to be radiolabeled and, consequently, their applications in biomedicine. This unexplored territory poses a significant research challenge that awaits investigation by the scientific community.

## 6. Toxicity Perspectives of MXene Nanoconjugates

Recent advances in MXene-based composites are quite exciting because of the potential results in the therapy and diagnosis of many ailments. Nevertheless, to advance clinical interpretation, several difficulties must be overcome. A significant challenge in utilizing these composites for cancer treatment lies in the lack of standardized parameters for safety testing. For safety research, numerous cell lines, dosages, and animal models have been used in diverse studies. Moreover, because diverse nanocomposites are fabricated using various MXenes under different chemical conditions and configurations, comprehensive research is required to better understand the relationships between the respective conjugate particles and MXenes.

Only a few studies have been published on the biocompatibility of MXenes. Table 2 details the toxicity effects of different MXene conjugates. MXenes have been significantly researched in terms of their biodistribution, clearance and accumulation characteristics, and cellular absorption. Because of their variable shape, acceptable biocompatibility, and strong physiological stability, MXenes have outstanding clinical translation capabilities [145,146]. Researchers evaluated the biosafety of MXene (Ti_3_C_2_T_x_) in an embryonic zebrafish model by examining its in vivo hazardous potential in a recent study. The authors examined the effects of MXenes on movement and the nervous system. MXene-treated embryos displayed normal movement with no alterations in neuron number, as observed in locomotion and neurotoxicity tests. Ti_3_C_2_T_x_ MXenes have no toxicological impact on muscular or neural activity. The authors determined that MXene had no neurotoxicity or ecotoxicity. The toxicological properties of MXene were assessed in early stage embryos. MXenes may have a negative impact on the initial stages of development since 46% of MXene-treated embryos died within 1–5 days after exposure. After 5 days of incubation, angiogenesis of the embryonic chorioallantoic membrane was impeded, indicating the potential toxicity of these structures during the early stages of development [147]. However, additional research is required to address the associated toxicity processes, as well as other critical elements of long-term biosafety, biodegradability, biocompatibility, dispersibility, and solubility [148]. Han and coworkers synthesized a 2D MXene-based nanocomposite of soybean phospholipid (SP) for the treatment of cancer via photo/chemotherapy. The as-prepared Ti_3_C_2_-SP nanocomposite showed no significant acute toxicity and exhibited good histocompatibility [149]. MXenes are typically eliminated from the body through feces and urine, excreting a total of approximately 10.35%. SP-functionalized MnO_x_/MXene (Ti_3_C_2_) nanocomposites exhibited increased stability as well as strong biocompatibility and dispersibility, suggesting promising prospects for clinical translation [149].

To be classified as “practically nontoxic”, nanocomposites must undergo comprehensive biosafety evaluations using animal models. These evaluations are crucial for understanding the acute and chronic effects of these agents on various organs. However, conjugating MXenes with polymers, proteins, and other nanomaterials can drastically modify their toxicity profile. The inclusion of biocompatible polymers or proteins can protect the reactive surface groups of MXenes, decreasing their cytotoxicity or immunogenicity. Furthermore, conjugation with targeting ligands or functional groups enables the targeted distribution of MXene-based nanomaterials to specific cells or tissues, reducing off-target effects and overall toxicity. In addition, this modification improves the stability of MXene-based nanomaterials, lowering the degradation and potential toxicity caused by the release of free MXene particles or ions. Although the overall effects of MXene composites on cancer therapy, tissue healing, and infection therapy are well known, the molecular processes underlying these reactions remain poorly understood. Close collaboration with molecular scientists is essential for fully comprehending specific therapeutic processes. The development of MXene composites is still in its early stages, necessitating research on the creation and utilization of extremely small quantum dots made of MXenes for treatment and regeneration.

## 7. Clinical Translations: Chances and Challenges

**Figure 9:**
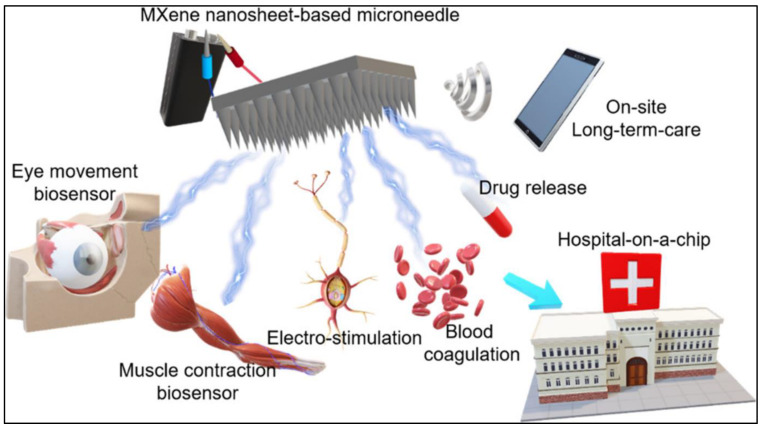
The concept of “hospital-on-a-chip” from the MXene-based microneedle system and its versatile applications. Reproduced with permission from [158]. Copyright 2021, American Chemical Society.

Owing to their distinct physicochemical characteristics, biocompatibility, and ease of functionalization, inorganic 2D MXene nanoplatforms have exhibited outstanding potential in the biomedical field compared to conventional organic materials. Their prospective applications include bioimaging, drug delivery, and biosensing. To find entry into clinical studies, MXene-based systems are being explored for their versatile biomedical applications. In this context, a team of researchers [158] developed a “hospital-on-a-chip” that utilizes advanced MXene nanosheets to create multifunctional microneedle electrodes for electrostimulation together with biosensing. The microneedles were composed of several small needles that may be employed for medication administration or biosensing (Figure 9). Wearable 2D MXene-incorporated microneedles could detect differences in the electric potential produced by eye movements or muscle contractions even when a person closes his or her eye. This makes them useful for monitoring disorders involving neuromuscular irregularities such as myasthenia gravis. These microneedles have the potential to become an integral part of the hospital-on-a-chip system, providing real-time examination of a patient’s vital signs and health parameters. This technology allows healthcare providers to continuously monitor patients without the need for invasive procedures, reducing the risk of infection and improving patient comfort. Moreover, the use of wearable biosensors can help detect diseases at an earlier stage, leading to better treatment outcomes.

However, there are challenges in implementing practical translation due to the potential nonspecific hazards of 2D MXene nanomaterials, which could pose risks to their safe clinical application [159]. Consequently, a comprehensive estimation of the harmfulness and compatibility of these materials with living organisms is necessary. Although short-term trials indicate biocompatibility, long-term safety assessments are needed to determine the potential specific neurotoxic effects on offspring [160]. Studies have explored surface modification strategies, such as collagen-based modifications, to enhance selectivity, minimize adverse effects on healthy cells, and reduce toxicity. Precise regulation of drug release is crucial for optimizing therapeutic outcomes while minimizing damage to healthy cells. This is achieved by the utilization of pH and NIR induction approaches. The characteristics of MXenes can be altered by meticulously crafting their composition and dimensions and performing surface functionalization. Enhancing the biomedical applications of MXenes can be achieved by investigating other morphological aspects, such as nanotubes and nanocages, and by combining MXenes with various functional materials to generate hybrids. Increasing the scale of MXene production is essential for clinical translation. Resolving these issues is crucial for maximizing the promise of MXenes in biomedical applications and advancing their widespread clinical acceptance.

## 8. Conclusions and Future Standpoints

Over the last few decades, researchers have been looking for perfect nanomaterials with characteristics such as high biocompatibility and tailored qualities for cancer therapeutic applications. MXene conjugation research in cancer theranostic applications is still in its early stages. The simplicity of the manufacturing process and the diverse alternatives available for surface functionalization of nanostructures for particular applications are critical factors for effectively exploring the biological characteristics of these nanoconjugates. These manufacturing processes will gain traction if integrated biological approaches are quicker, more economical, and more environmentally friendly. Based on the available research, there is no practical and acceptable green synthesis procedure for the manufacture of MXenes. This feature requires special attention since creating an effective and practical synthesis technique can broaden the use of MXenes for cancer theragnostic benefits. In vitro cell line evaluations are an essential aspect of researching the biomedical impacts of nanoformulations. In vitro evaluations of developed MXene conjugates for tumor therapeutic and diagnostic benefit are highly important because they provide early indications of anticancer activity, biodegradation characteristics, and apoptotic mechanisms such as oxidative stress and, primarily, of the effect of these nanoconjugates on normal cells. According to recent findings, the cytotoxic properties of MXene-based conjugates require further exploration. MXene conjugates developed for cancer therapy must be aggressive toward cancer cells while being harmless to healthy tissues and cells. We expect that this topic will be extensively examined in future studies to provide a clear picture.

Following successful cell line experiments, the theragnostic activity of these nanoconjugates should be evaluated in live animals to prove their biocompatibility. These animal models are critical because they provide a good picture of the effectiveness of MXene conjugates against tumor cells and because of the many adverse consequences they might cause in the body after treatment. Furthermore, the biocompatibility and excretion mechanisms of MXene conjugates must be explained to rule out any adverse reactions they may have in the body due to their in vivo biodistribution. These elements must be given special consideration because they offer useful insight into the use of MXene conjugates in different cancer theragnostic applications, such as photothermal treatment, multimodal imaging, and combination therapy, in addition to other traditional therapeutic techniques. Clinical trials are important for evaluating the biocompatibility and anticancer properties of MXene conjugates. Unfortunately, the use of MXene-conjugated cancer theranostics has yet to reach this stage. Because malignancy treatment continues to constitute one of the most challenging parts of the health care industry, this component of the investigation of MXene conjugates must be properly explored and addressed as soon as possible. Promising in vitro and in vivo experiments with outstanding results have been described in the literature, providing vital insights into the development of an effective multifunctional MXene platform. There is a wealth of research on phytochemicals with interesting anticancer potential that may be studied utilizing the drug delivery effectiveness of MXenes. To advance these findings to clinical trials, the issue of cytotoxicity must be extensively investigated.

Despite major advances in cancer research, cancer management remains challenging, particularly in the later stages of the disease. Different kinds of pathophysiological mechanisms related to cancer and metastatic routes are important impediments that render traditional treatment techniques ineffective. The application of nanotechnology for treating lethal illnesses such as cancer is critical. Using the unique features of different nanomaterials, innovative biomedical devices, such as those for drug delivery, imaging, and sensing probes, have been effectively constructed. MXene conjugates are a unique kind of approach that are still in their early stages of use in cancer treatment for medication delivery and diagnostic purposes. Significant progress has been made by researchers worldwide in researching alternative functional groups and fabricating MXene conjugates for particular tumor types and tactics, with encouraging outcomes. Efficient utilization of MXenes in conjunction with imaging modalities can yield excellent findings in cancer diagnosis, paving the way for substantial advances in oncology research.

## Figures and Tables

**Table 2:** Toxicity effect of different MXene conjugates.

MXene	Composite Particle	Delaminating Agent	Etching Agent	Dose	Cell Line Used	Toxicity	Reference
Ti_3_C_2_	Au	Ultrasound	LiF-HCl mixture	1 to 200 μg mL^−1^, 48 h	HaCaT, A375	Approximately 50% of the cells were still viable at concentrations greater than 50 µg mL^−1^	[150]
Ti_3_C_2_	SP MnO_x_	TPAOH	HF	10 to 160 μg mL^−1^, 48 h	4T1	At 160 μg mL^−1^, no substantial toxicity was identified, indicating acceptable biocompatibility	[151]
Ti_3_C_2_	Cellulose	TPAOH	HF	78.4–313.6 ppm	HepA1-6, SMMC-7721, HepG2, U-118MG	Integration into the hydrogel decreased the toxicity as compared to the dispersed MXene solution	[79]
Nb_2_C	MSN	Ultrasound	HF	18.75–300 μg mL^−1^, 24 h	U87	No notable toxicity with the formulation Observed CTAC-mediated toxicity	[152]
Ti_3_C_2_	Chitosan	Ultrasound	HF	0–300 μg mL^−1^, 24 h	HeLa	No notable toxicity	[153]
Ti_3_C_2_	Chitosan/HA hollow microcapsules Au	TPAOH	HF	0–100 μg mL^−1^, 24 h	MCF-7	Concentration dependent toxic effect The increase in cytotoxicity with concentration indicates chemotherapy activity	[154]
Ti_3_C_2_	PVP	DMF	HF	50 and 500 μg mL^−1^, 24 h	293T, MCF-7	100% cell viability at all concentrations	[155]
Ti_2_N	SP	Ultrasound Higher temperature	KF-HCl mixture	0–80 μg mL^−1^, 24 h	293T, 4T1, U87	Excellent biocompatibility at 80 ppm, with no cytotoxicity Complete tumor cell eradication was seen under 808 and 1064 nm NIR radiation	[156]
Ta_4_C_3_	SP MnO_x_	Ultrasound	HF	25–400 μg mL^−1^, 48 h	4T1	4T1 cells displayed toxicity to 808 nm irradiation	[77]
Nb_2_C	PLL	Ultrasound and TBAOH	HF	500 μg mL^−1^, 48 h	HaCaT, A375	Exhibited a dose- and cell-mediated toxicity	[157]

## Data Availability

Data are contained within the article.
